# Nicole Redvers: bridging the many worlds of human and planetary health

**DOI:** 10.2471/BLT.25.031125

**Published:** 2025-11-01

## Abstract

Nicole Redvers talks to Gary Humphreys about integrating Indigenous medicine and planetary health into global health discourse.

*Q: You grew up in a small Dene First Nation community. How did that upbringing shape you as a person and as the healer and thought leader you have become?*

A: I grew up deeply connected to my community and to the land – by which I mean the natural world around me. We were around 300 people back then. I grew up in the log house that my father had built, surrounded by boreal forest near Great Slave Lake. It is too cold for agriculture up there and we couldn’t cultivate crops, so my family were hunters and fishers. We hunted moose, caribou, sometimes buffalo, and fished. We also gathered berries and medicinal plants.

*Q: So you were exposed to medicinal plant knowledge from a young age?*

A: Absolutely. It was part of our everyday reality. We had only a small nursing station nearby and there were very real barriers to seeking health care in the formal system. I saw friends and family members avoid seeking care because of fear of discrimination or negative experiences. Also, you have to remember that up until very recently, Indigenous medicine was the primary system.

*Q: What kinds of medicines were available to you?*

A: There are protocols around what can be shared publicly, so I cannot talk about that. But I can say that berries were commonly used, and that much of our medicinal knowledge also comes from animals. For chronic and sub-acute conditions, our traditional system was and remains highly effective. 

*Q: How did you come to embrace naturopathy?*

A: When I reached school age, my parents moved us to a larger town, on another First Nation’s territory, so that I could attend school without having to live in a boarding home. That was a big change for us, and like many teenagers, I drifted toward a more “modern” lifestyle. The turning point came during university when I accidentally walked into a talk on naturopathic medicine. I say accidentally, but I’m not sure it was really by chance. Our Elders always tell us that we're supposed to be in the places that we are, and that it's up to us to take the opportunities that are presented. So, I think at some level I was meant to walk in on that lecture. In my home region, we had only Western medicine and Indigenous traditional medicine – I didn’t even know naturopathic doctors existed. The idea that you could integrate both traditions within a standard medical framework was eye-opening. The very next day, I switched my degree path.

“Indigenous systems are inherently holistic.”

*Q: Why did naturopathy resonate so deeply with you?*

A: Because it integrates modern scientific knowledge with traditional and natural approaches to healing. It is also grounded in core principles such as treating the whole person, addressing root causes, supporting the body’s innate healing capacity, and emphasizing prevention, and it combines conventional medical training with therapies like clinical nutrition, botanical medicine, acupuncture, physical treatments and lifestyle counselling. It evolved from Euro-Western traditions but it now incorporates elements from many traditional systems, including Indigenous traditions. 

*Q: You are often described as someone who works to bridge different knowledge systems. What does that involve?*

A: For me, it starts with building relationships and respect. It is not about merging the different traditions into one system. The goal is to apply what is needed in each context. Sometimes it is mostly Indigenous knowledge, other times a balanced integration. At the Arctic Indigenous Wellness Foundation, which was founded in 2017 in Yellowknife, Canada, culturally grounded land-based wellness programmes are offered alongside support for individuals navigating the formal health system. Its urban healing camp, which is located in the centre of the city, provides traditional ceremonies, counselling, and community supports addressing trauma, addiction and social inequities while fostering holistic, culturally safe care in dialogue with mainstream medical services. Another part of my work is knowledge translation, helping different systems understand each other. 

*Q: But if your protocols forbid the sharing of information about your medicines, how can knowledge be exchanged?*

A: Well, first, it is important to acknowledge that there are many Indigenous traditions and an incredible diversity of Indigenous Peoples and medicine systems worldwide. Some are more open than others, and the marketplace is filled with substances – ranging from nutritional supplements to pharmaceuticals – claiming to derive from traditional knowledge systems. Many Indigenous communities do not openly disclose specific knowledge due to concerns about intellectual property and cultural respect. Our protocols, which prohibit commercialization, help protect our medicines. That said, there has been some movement in this area. For example, WIPO (World Intellectual Property Organization) now requires companies to acknowledge patents based on Indigenous knowledge, but consent is still not required. More needs to be done. For their part, many Indigenous communities are just beginning these conversations. It will take time, and different communities will have different views. We are having internal discussions and are developing a grant to explore how climate change and emergencies might shift thinking on protocol around knowledge sharing with regard to medicines, for example. But I would also say that knowledge sharing is not just about knowledge of active ingredients. It is about ensuring that Indigenous Elders and knowledge keepers are part of health system conversations, research teams, and curriculum development. It also means creating space for Indigenous healing practices to be offered alongside Western care in ways that are safe and supported by communities. I spend a lot of time mentoring young Indigenous health professionals on how to navigate this interface without feeling they have to abandon their cultural identities. 

*Q: In practical terms, what changes are needed within mainstream health systems to better serve Indigenous populations and support knowledge integration?*

A: First, we need structural change, embedding Indigenous governance and leadership into health systems, not as an afterthought but as a core principle. That includes reforming medical education to equip providers with cultural humility, understanding of Indigenous rights, and relational competencies. We also need to ensure that Indigenous communities have the resources and autonomy to lead their own health initiatives, whether that’s through land-based healing, community health programmes, or research. Finally, data governance is key: Indigenous Peoples must have control over how health data are collected, interpreted and used.

“Our traditional system remains highly effective.”

*Q: Why is it important to build bridges between the different traditions?*

A: There’s a huge opportunity and an urgent need to value relational ways of knowing in global health. Indigenous systems are inherently holistic; they view health as interconnected with land, community and spirit. That perspective is vital as we grapple with planetary health crises and systemic inequities. 

*Q: How does planetary health resonate with Indigenous perspectives?*

A: My Indigenous planetary health-related work bridges internationally-situated Indigenous networks addressing climate and ecological justice through a health lens. For many Indigenous Peoples, the concept of planetary health is not new; we’ve always understood that human health is inextricably linked with the health of the land, water and ecosystems. And like everyone else, we are well aware of the impacts of climate change. For example, the recent wildfires in Canada have destroyed many of our traditional harvesting areas. Many communities in the short term have therefore lost connections with some of their lands. Despite this, the Indigenous perspective offers a way to reconnect, to restore an ecological sense of belonging that is still present in Indigenous worldviews regardless of crisis. What planetary health offers is a language and platform that can elevate Indigenous voices within global discourse. Through the networks, we aim to centre Indigenous leadership in addressing climate and ecological justice, and to build partnerships that are rooted in reciprocity and respect for Indigenous knowledge systems. 

*Q: You serve on WHO’s Technical Advisory Group on Ethics and Climate Change. Do your discussions there give you hope for the future?*

A: It is encouraging to see a WHO-convened group discussing health and climate change as part of the broader health ethics discussion. Especially since it is a discussion that includes consideration of planetary health – a solutions-oriented, transdisciplinary approach that addresses how planetary impacts affect human and ecological health. There is also recognition that Indigenous knowledges offer valuable insights for finding better paths forward. In other spaces, notably the pharmaceutical market place, there is still a tendency to view Indigenous knowledge as something to be commercialized or commoditized. 

*Q: What role can international bodies like WHO play in further advancing agendas that bridge different health-care traditions?*

A: WHO and other global health organizations can play a critical role in recognizing and supporting Indigenous-led solutions. This means going beyond token inclusion to actively partnering with Indigenous networks in shaping global health policies. WHO can also foster dialogue between Indigenous knowledge holders and scientists to co-create models of care that are both culturally grounded and evidence-informed. I see a rising generation of Indigenous scholars, healers and advocates who are bringing their voices to the forefront of global discussions. There is growing recognition that Indigenous knowledge systems go beyond cultural preservation and provide valuable insights for creating sustainable futures. 

